# *Tuber indicum* shapes the microbial communities of ectomycorhizosphere soil and ectomycorrhizae of an indigenous tree (*Pinus armandii*)

**DOI:** 10.1371/journal.pone.0175720

**Published:** 2017-04-14

**Authors:** Qiang Li, Jian Zhao, Chuan Xiong, Xiaolin Li, Zuqin Chen, Ping Li, Wenli Huang

**Affiliations:** 1 Biotechnology and Nuclear Technology Research Institute, Sichuan Academy of Agricultural Sciences, Chengdu, Sichuan, China; 2 College of Life Science, Sichuan University, Chengdu, Sichuan, China; 3 Soil and Fertilizer Institute, Sichuan Academy of Agricultural Sciences, Chengdu, Sichuan, China; Friedrich Schiller University, GERMANY

## Abstract

The aim of this study was to investigate the effect of an ectomycorrhizal fungus (*Tuber indicum*) on the diversity of microbial communities associated with an indigenous tree, *Pinus armandii*, and the microbial communities in the surrounding ectomycorhizosphere soil. High-throughput sequencing was used to analyze the richness of microbial communities in the roots or rhizosphere of treatments with or without ectomycorrhizae. The results indicated that the bacterial diversity of ectomycorhizosphere soil was significantly lower compared with the control soil. Presumably, the dominance of truffle mycelia in ectomycorhizosphere soil (80.91%) and ectomycorrhizae (97.64%) was the main factor that resulted in lower diversity and abundance of endophytic pathogenic fungi, including *Fusarium*, *Monographella*, *Ustilago* and *Rhizopus* and other competitive mycorrhizal fungi, such as *Amanita*, *Lactarius* and *Boletus*. Bacterial genera *Reyranena*, *Rhizomicrobium*, *Nordella*, *Pseudomonas* and fungal genera, *Cuphophyllus*, *Leucangium*, *Histoplasma* were significantly more abundant in ectomycorrhizosphere soil and ectomycorrhizae. Hierarchical cluster analysis of the similarities between rhizosphere and ectomycorrhizosphere soil based on the soil properties differed significantly, indicating the mycorrhizal synthesis may have a feedback effect on soil properties. Meanwhile, some soil properties were significantly correlated with bacterial and fungal diversity in the rhizosphere or root tips. Overall, this work illustrates the interactive network that exists among ectomycorrhizal fungi, soil properties and microbial communities associated with the host plant and furthers our understanding of the ecology and cultivation of *T*. *indicum*.

## Introduction

Truffles, belonging to the *Tuber* genus (Ascomycota, Pezizales), are ectomycorrhizal fungi (ECMF) that produce hypogeous fruiting bodies [1, 2] that are highly prized as a food delicacy. Truffles can form a symbiotic relationship with trees of several genera, including *Quercus*, *Corylus*, *Pinus* and *Abies* [3–6]. The black truffle (*Tuber melanosporum*) and the white truffle (*T*. *magnatum*) are highly valued on the European market because of their unique flavors [7–9]. *T*. *indicum*, commonly known as the Chinese black truffle and one of China’s major commercial species [10, 11]. shares similar morphological characteristics and has a close phylogenetic relationship with *T*. *melanosporum*. Polysaccharides and ribonuclease isolated from its fruiting bodies and fermentation system showed a strong antitumor and antiproliferative activity [12, 13]. *T*. *indicum* forms typical ectomycorrhizae structures in Chinese indigenous plants, such as *Castanea mollissima* and *Pinus armandii*, and ectomycorrhizae has been successfully synthesized in China [11, 14]. The synthesis and artificial cultivation of ectomycorrhizae have attracted more and more attention due to the decrease in wild tuber yield, which is believed to be the result of vegetation destruction, forest fires, improper harvesting and other human factors.

Ectomycorrhizal fungi play an important role in ecosystems. Truffle species often form brûlé (an area devoid of herbaceous cover) around a host tree, and reduce biodiversity of bacteria and fungi in the brûlé area [15, 16]. Truffle, when dominant, is predicted to affect associated soil fungal communities [16–18], which may lead to the formation of this “brûlé” region around the base of its host. It is also known that the microbial communities associated with truffle grounds vary by season and by region, for different truffle species [19–22]. Different *Tuber* spp. may exert different competitive effects on other mycobionts. Ectomycorrhizae can be beneficial to plant productivity by enhancing plant growth or resistance to abiotic stress [23–26]. Ectomycorrhizal fungi can improve nitrogen and water acquisition of host plants, playing a key role in the nutrition of forest trees [27–29]. The colonization of ectomycorrhizal fungi causing higher soil porosities, which has proven to play a crucial role in achieving success in black truffle plantations [30]. Nevertheless, some indirect effects of the truffle ectomycorrhizal symbiosis have not been investigated, such as the effects on other soil microbes, and how the endophytic bacteria of host plants may interact with the mycorrhizal synthesis and affect plant growth.

Although cultivation of truffles has been attempted for decades, the frequent failure to develop productive plantations indicates that the reproduction, cultivation and conditions stimulating ascocarp formation remain mysterious. Endophytes and soil microbes play an important role in the growth and development of the plant host [31–34]. They can participate in the metabolic processes of the host, produce biological molecules that have growth-promoting or antibacterial activities, and ultimately affect the yield and quality of the host [35–38]. The effects of wild truffles on soil microorganisms in the surrounding soil has been studied [22, 39], however, it is unclear what role environmental factors play in the composition of these microbial communities. In this study, we established a controlled artificial mycorrhizal synthesis system with *T*. *indicum* and *Pinus armandii* with the goal of testing how the ectomycorrhizal symbiotic relationship affects microbial diversity and the communities associated with ectomycorrhizae and the soil surrounding the host plant during the early symbiotic stage. We also analyzed changes in soil properties and their correlation with microbial communities to lay a foundation for artificial cultivation of *T*. *indicum* and reveal the microbial interactions among ectomycorrhizal fungi, surrounding soil communities and the host plant.

## Materials and methods

### Sampling strategy and soil analyses

**Fig 1:**
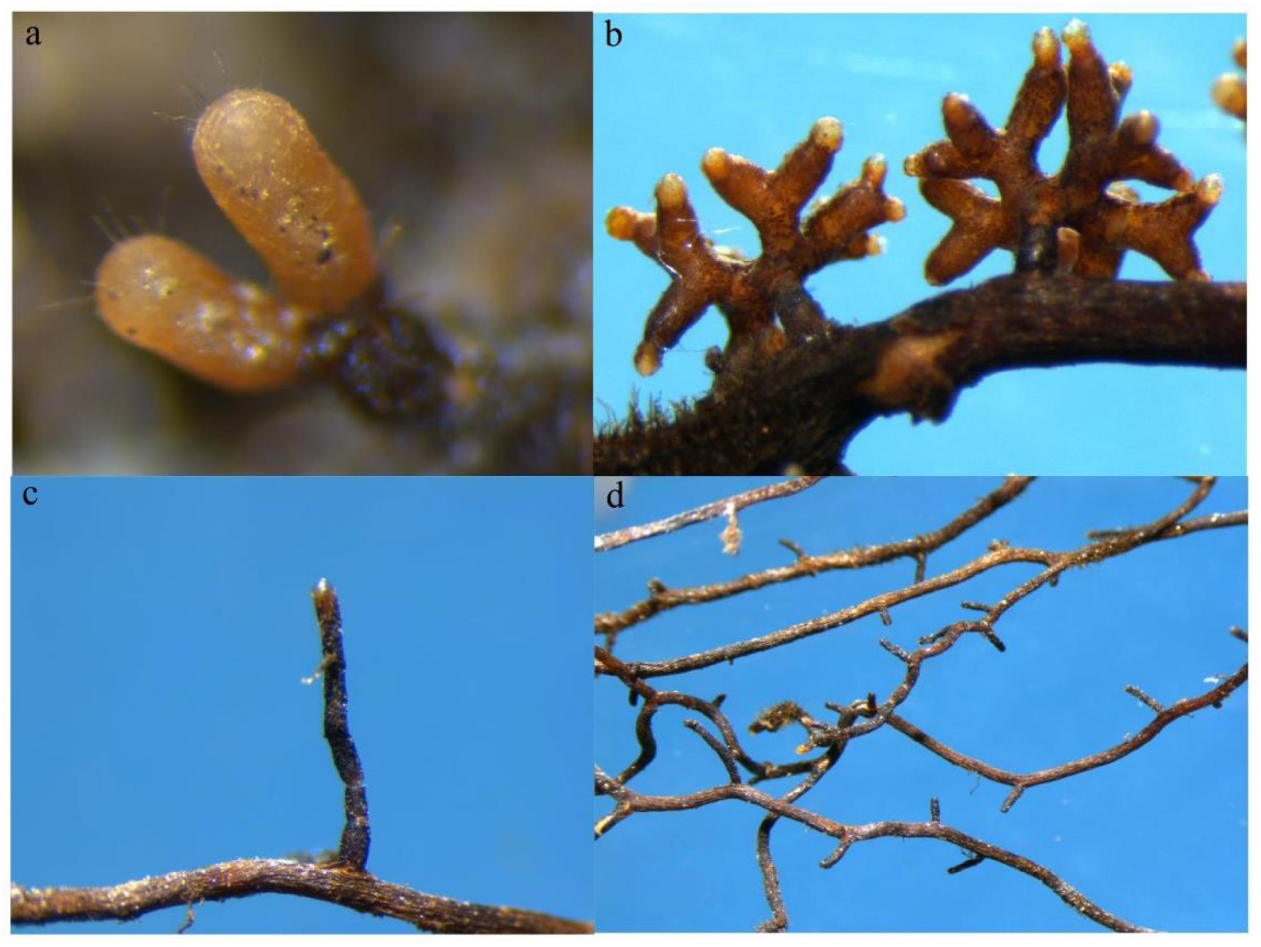
Root tips of *Pinus armandii* in association with *Tuber indicum* (a and b) or without *T*. *indicum* (c and d).

In southwest China, *Tuber indicum* fruiting bodies were found most frequently in the soil surrounding the indigenous plant, *Pinus armandii*. To reveal the effects of ectomycorrhizal fungi on rhizosphere soil and the microbial community associated with host plant, the ectomycorrhizae of *T*. *indicum* and *P*. *armandii* were artificially synthesized in greenhouses. *P*. *armandii* seeds were purchased from a commercial company. Seeds were surface sterilized with 30% H_2_O_2_ for 4 h and washed three times with distilled water [14]. Surface-sterilized seeds were sown in a plastic container filled with sterilized substrates, which were autoclave sterilized for 90 min at 121°C, (vermiculite, perlite and water at a ratio of 1:1:1, v/v/v) to germinate. After one month, seedlings were transplanted into container with 1 L sterilized substrate (peat, vermiculite, organic soil and water at a ratio of 1:1:1:0.9, v/v/v/v). The final pH of the homogenized substrate was adjusted to 7.5 by adding calcium hydroxide. Spore powder was obtained by blending ascocarps [14] (collected from the *P*. *armandii* forest and identified by morphological and molecular analysis) that had been surface sterilized with 75% alcohol, and soaked with sterile water to incite the spores to be released and germinate. We inoculated 2 g of this spore powder into the substrates. Three *P*. *armandii* seedlings that were not inoculated with truffle spores served as controls. All pots (each treatment had three replicates) were maintained in the greenhouse under the same conditions. Plants were watered every three days. No fertilizers were added to the plants [14]. After five months, plant root tips and soil were harvested. Morphological and molecular analysis of the mycorrhiza were performed by microscope and ITS-rDNA sequence analyses (Fig 1, GenBank accession number KY296094). Mycorrhization were successfully obtained in all 3 of the seedlings inoculated with truffle spores, and another 3 control seedlings were not colonized by Truffles as molecular and morphological analysis revealed. *P*. *armandii* roots, mycorrhized with truffle mycelia (ECM) or not colonized (CK), were surface sterilized, and separately with their surrounding soil, were extracted for their DNA for high throughput sequencing. The properties of soil samples around the roots were analyzed according to our previously described method [40]. Briefly, Soil particle size distribution was determined using the pipette method. pH was measured in soil water extracted by dissolving air-dried soil in distilled water at a ratio of 1:5. Organic matter content was estimated using the Tyurin method. Total nitrogen was determined by the Kjeldahl method. To evaluate differences in the physical and chemical soil properties, we applied a hierarchical cluster analysis to the data set (SPSS v. 19.0) and used the between-groups linkage method. Root tips of *P*. *armandii* mycorrhized with *T*. *indicum* were assigned to ECM (ectomycorrhiza) and the surrounding soils were assigned to ECM.S. Roots from the control trees lacking the *T*. *indicum* partner were assigned to CK and soils were assigned to CK.S. All experiments were conducted in triplicate.

### Surface sterilization and DNA Extraction

The mycorrhizae or control roots of *P*. *armandii* were washed with distilled sterile water. Their surfaces were sterilized by soaking the tissues sequentially in 75% ethanol for 3 min, in 3% sodium hypochlorite for 2 min, and in 75% ethanol for 3 min, followed by a rinse with sterile water [40]. The effectiveness of surface sterilization was verified by plating 100 μL of the final rinse on luria broth (LB) plates and incubating them at 28°C for 48 h. Genomic DNA of the tissues and endophytes were extracted using hexadecyl trimethyl ammonium bromide (CTAB) method. DNA of soils around the roots was extracted using the Soil DNA Kit (D5625-01, Omega Bio-tek Inc., Norcross, GA, USA) in accordance with the instructions. DNA concentration and purity were monitored on 1% agarose gels. According to the concentration, DNA was diluted to 1 ng/μL using sterile water.

### HiSeq sequencing

All samples 16S V4 and ITS1 genes were amplified using the universal primers 515F-806R and ITS5-1737F with the barcode as a marker for distinguishing samples respectively [21]. All PCR reactions were carried out with Phusion^®^ High-Fidelity PCR Master Mix (New England Biolabs, Ipswich, MA, UK). PCR was conducted according to Li et al [40]. PCR products were mixed in equidensity ratios. Then, mixture PCR products were purified with Qiagen Gel Extraction Kit (Qiagen, Germany). Sequencing libraries were generated using TruSeq^®^ DNA PCR-Free Sample Preparation Kit (Illumina, USA) following manufacturer's recommendations and index codes were added. The library quality was assessed on the Qubit@ 2.0 Fluorometer (Thermo Scientific) and Agilent Bioanalyzer 2100 system. The library was sequenced on an Illumina HiSeq 2500 platform and 250 bp paired-end reads were generated [41].

### Pyrosequence date analysis

Paired-end reads was assigned to samples based on their unique barcode and truncated by cutting off the barcode and primer sequence. Reads that overlapped, which were generated from the opposite end of the same DNA fragment, were merged using FLASH [42]. Quality filtering on the raw tags was performed under filtering conditions that were selected to obtain the high-quality clean tags [43] according to the QIIME [44] quality controlled process. The tags were compared with the reference database using the UCHIME algorithm [45] to detect and remove chimera sequences [46]. Sequence analysis was performed using the Uparse software [47]. Sequences with ≥97% similarity were assigned to the same OTUs. A representative sequence for each OTU was screened for further annotation. For each representative sequence, the GreenGene Database [48] was used based on RDP 3 classifier [49] algorithm to annotate taxonomic information. OTU abundance information was normalized using a standard of sequence number corresponding to the sample with the fewest sequences. Subsequent analysis of alpha diversity and beta diversity were all performed based on this output normalized data. Alpha diversity is applied in analyzing complexity of species diversity for a sample through 6 indices, including Observed-species, Chao1 (http://www.mothur.org/wiki/Chao), Shannon, Simpson, ACE (http://www.mothur.org/wiki/Ace), Good-coverage. All this indices and beta diversity in our samples were calculated with QIIME (Version 1.7.0). Raw reads were submitted to Sequence Read Archive (SRA) database (accession number: SRR5278654-SRR5278665).

### Statistical analysis

Data of this study are presented as means ± standard deviation (SD) of three biological triplicates for each treatment. Statistical analysis was carried out by one-way analysis of variance (ANOVA) using SPSS 19.0. Least significant difference (LSD) was performed to test if the ANOVA result between different treated groups was significant at P < 0.05.

## Results

### Bacterial diversity indices

To determine the composition of bacterial communities in the ectomycorrhizae and in the surrounding soil, we carried out 16S V4 rRNA barcoded pyrosequencing. 45,077–79,441 reads were obtained per sample after quality control procedures (S1 Fig). Altogether, 41 phyla, 81 classes, and 439 genera of bacteria and archaea, with between 1184–2084 OTUs were detected at the 97% similarity threshold. There were no significant differences in the numbers of observed species among different samples (Table 1). Other community richness indices, such as chao1 and ACE, also showed the same pattern for all samples. Two indices representing community diversity, the Shannon and Simpson indices, indicated that bacteria was most diverse in the CK.S treatment, and that the CK treatment had the least diverse bacterial community. The bacterial diversity of ectomycorhizosphere soil was significantly decreased compared with CK.S (P < 0.05). While the Simpson index of ECM was significantly higher than CK (P < 0.05), their Shannon index did not differ significantly.

**Table 1:** Community richness and diversity indices of bacteria associated with *Pinus armandii* roots and surrounding soils with or without *Tuber indicum* partner.

Sample name	Observed species	shannon	simpson	chao1	ACE	Goods coverage	PD whole tree
**ECM.S**	1353.33±172.79a	7.48±0.25b	0.98±0.00b	1494.50±183.64a	1523.40±189.14a	0.99±0.00a	90.39±4.79ab
**CK.S**	2084.67±374.68a	8.97±0.25a	0.99±0.00a	2339.51±492.21a	2360.18±501.44a	0.99±0.00a	129.53±16.79a
**ECM**	1514.67±425.14a	7.38±0.21bc	0.98±0.00b	1693.01±452.52a	1729.36±483.90a	0.99±0.00a	102.86±17.92ab
**CK**	1184.00±111.14a	6.50±0.30c	0.95±0.00c	1314.53±463.51a	1329.81±122.19a	0.99±0.00a	88.37±12.91b

ECM and ECM.S, ectomycorrhizae (*Pinus armandii* mycorrhized with *Tuber indicum*) and ectomycorrhizosphere soil. CK and CK.S, roots and soils from cultivated *P*. *armandii* without *T*. *indicum* partner. Each value is the mean of 3 replicates (±SD). Values followed by different lowercase letters indicate significant differences (P < 0.05) between samples in a line.

### Fungal diversity indices

About 38,052–73,338 qualified reads were obtained per sample (S1 Fig), representing 6 phyla, 21 classes, and 122 genera of fungi, with between 99–332 OTUs observed at a 97% similarity level. The number of fungal species observed in CK.S was significantly greater than in other samples (P < 0.05) (Table 2). Other community richness indices, such as chao1 and ACE, indicated the CK.S treatment had the fungal community with the highest richness, whereas the ECM treatment had the fungal community with the lowest richness. Shannon and Simpson indices also found the highest fungal diversity in the CK.S treatment and the lowest fungal diversity in the ECM treatment.

**Table 2:** Community richness and diversity indices of fungi associated with *Pinus armandii* roots and surrounding soils with or without *Tuber indicum* partner.

Sample name	Observed species	shannon	simpson	chao1	ACE	Goods coverage	PD whole tree
**ECM.S**	181.00±46.50b	1.84±0.37b	0.48±0.11b	215.40±61.26ab	229.24±64.69ab	1.00±0.00a	68.45±16.92b
**CK.S**	332.00±41.61a	4.12±0.55a	0.81±0.06a	352.96±39.11a	370.90±37.29a	1.00±0.00a	114.00±13.60a
**ECM**	99.67±14.41b	0.84±0.19b	0.28±0.11c	121.85±25.63b	131.77±17.41b	1.00±0.00a	39.38±4.68b
**CK**	181.33±27.38b	1.87±0.25b	0.59±0.06b	233.15±44.70ab	248.62±38.75ab	1.00±0.00a	70.94±13.46b

ECM and ECM.S, ectomycorrhizae (*Pinus armandii* mycorrhized with *Tuber indicum*) and ectomycorrhizosphere soil. CK and CK.S, roots and soils from cultivated *P*. *armandii* without *T*. *indicum* partner. Each value is the mean of 3 replicates (±SD). Values followed by different lowercase letters indicate significant differences (P < 0.05) between samples in a line.

### Characteristics of rhizosphere or ectomycorrhizosphere soil

The properties of soil samples around the roots were analyzed according to our previously described method [40]. Some physicochemical properties of the soil surrounding *P*. *armandii* root tips differed between treatments with or without *T*. *indicum* (Table 3). The pHs of the soil samples showed no significant difference, varying from 5.96 to 7.06. Ectomycorrhizosphere soil contained more sand than CK.S, indicating higher soil porosities. The content of total potassium and available copper was significantly higher in ECM.S compared with CK.S. Other properties, such as the organic matter, effective nitrogen, available phosphorus, available manganese, available calcium and available magnesium, were also a little higher in ECM.S compared with CK.S.

**Table 3:** Physical and chemical properties of *Pinus armandii* rhizosphere and ectomycorrhizosphere soil.

Sample	pH	Sand (%)	Silt (%)	Clay (%)	OM (g/kg)	TN (g/kg)	TP (g/kg)	TK (g/kg)	AN (mg/kg)	AP (mg/kg)	AK (mg/kg)	AFe (mg/kg)	AMn (mg/kg)	ACu (mg/kg)	AZn (mg/kg)	ACa (cmol/kg)	AMg (cmol/kg)
**CK.S1**	6.94±0.08a	36.62±0.87d	17.57±0.49b	43.85±0.70a	44.08±1.08c	2.04±0.02b	0.98±0.01c	14.88±0.03e	116.76±1.37b	29.28±0.54b	117.58±1.89c	26.17±0.31e	8.67±0.16cd	3.12±0.01b	1.58±0.05c	11.19±0.14b	1.48±0.03cd
**CK.S2**	5.96±0.07c	37.40±0.84cd	25.49±0.31a	35.14±1.09c	46.34±0.50abc	2.10±0.04ab	1.12±0.02b	14.96±0.01de	99.64±2.27c	31.01±0.27ab	119.29±2.01bc	26.68±0.04de	8.56±0.28d	2.50±0.02b	1.79±0.01bc	11.41±0.13b	1.30±0.02d
**CK.S3**	6.97±0.09a	33.75±0.40d	24.31±0.41a	39.97±0.70ab	44.38±0.82bc	1.75±0.01c	1.07±0.01b	15.28±0.06d	105.43±1.32c	31.18±0.26ab	131.94±2.14a	27.80±0.17bcd	8.62±0.11d	2.87±0.05b	1.60±0.05c	9.61±0.13c	1.55±0.07bc
**ECM.S1**	7.06±0.09a	45.29±1.07a	15.76±0.39b	36.99±0.92bc	48.73±1.15a	2.26±0.05a	1.09±0.02b	16.54±0.11b	131.43±1.68a	32.50±0.54a	112.76±0.13c	30.99±0.55a	10.24±0.21ab	3.38±0.01a	1.78±0.06bc	12.90±0.16a	1.78±0.06b
**ECM.S2**	6.70±0.08ab	40.69±0.57bc	24.25±0.36a	33.10±0.75cd	48.41±0.57ab	2.15±0.06ab	1.25±0.02a	15.81±0.15c	118.89±4.56b	32.47±0.27a	98.15±0.98d	27.74±0.22cd	9.50±0.15bc	3.37±0.03a	2.19±0.04a	12.86±0.10a	1.70±0.06bc
**ECM.S3**	6.51±0.08b	42.04±0.97ab	25.67±0.69a	30.32±0.81d	47.67±0.79abc	2.05±0.03b	1.23±0.01a	17.93±0.05a	124.60±1.24ab	31.68±0.58a	125.67±2.04ab	28.41±0.21bc	10.99±0.07a	3.32±0.04a	1.92±0.03b	11.60±0.18b	2.03±0.06a

OM, organic matter; TN, total nitrogen; TP, total phosphorus; TK, total potassium; AN, effective nitrogen; AP, available phosphorus; AK, available potassium; AFe, available iron; AMn, available manganese; ACu, available copper; AZn, available zinc; ACa, available calcium; AMg, available magnesium. CK.S, rhizosphere soil; ECM.S, ectomycorrhizosphere soil. Each value is the mean of 3 replicates (±SD). Values followed by different lowercase letters indicate significant differences (P < 0.05) between samples in a line.

**Fig 2:**
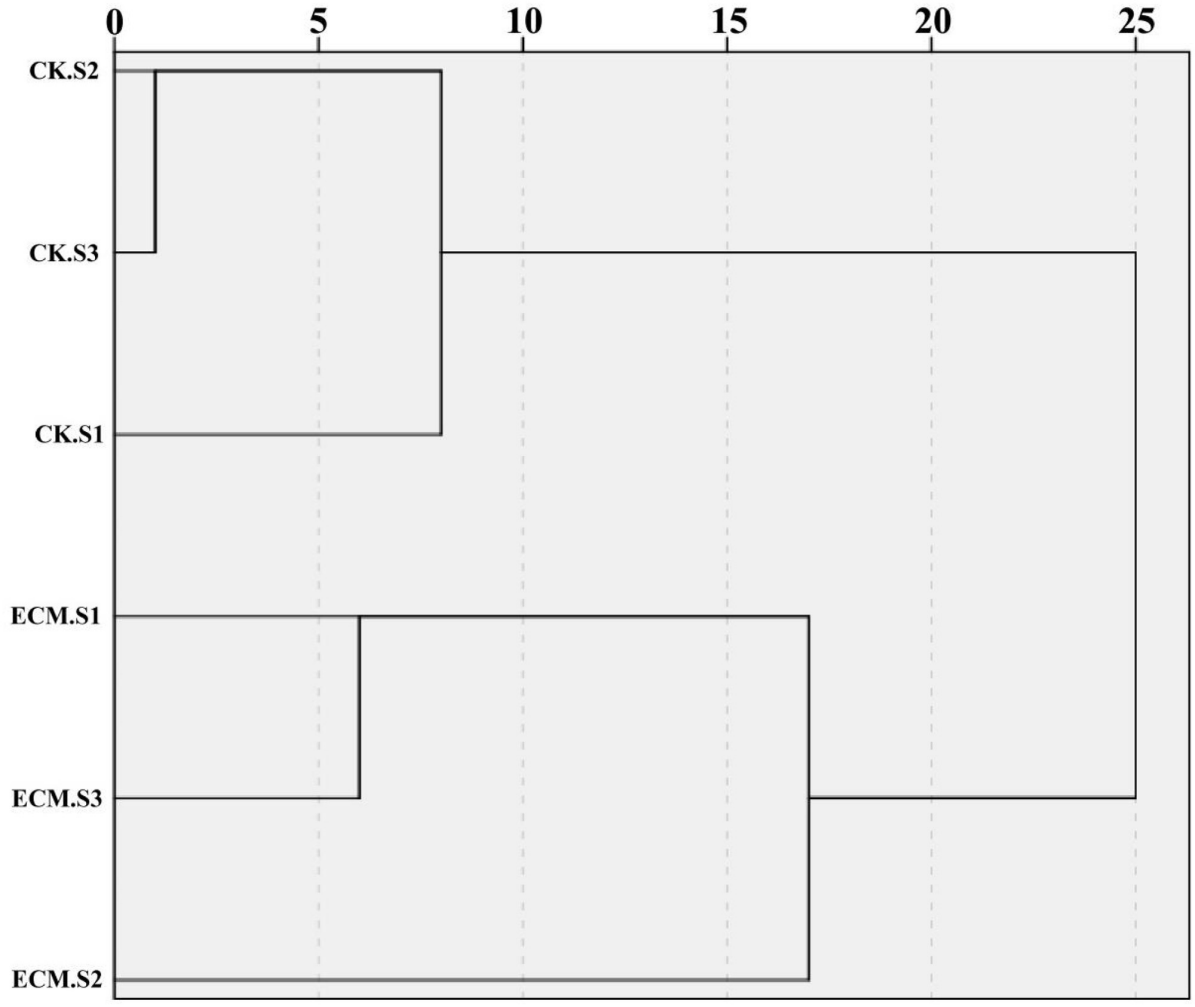
Hierarchical cluster analysis of the similarities among different samples based on the soil properties using the the between-groups linkage method in SPSS. CK.S, rhizosphere soil; ECM.S, ectomycorrhizosphere soil.

A cluster analysis dendrogram of the soil property similarities between different samples show two main clusters (Fig 2). The first cluster was rhizosphere soil and the second was ectomycorrhizosphere soil. These two treatments showed significant variability in soil properties, such as the organic matter, total nitrogen, available phosphorus, available calcium and available magnesium, indicating a feedback effect of ectomycorrhiza on the surrounding soil.

### Taxonomic analyses of bacterial communities

**Fig 3:**
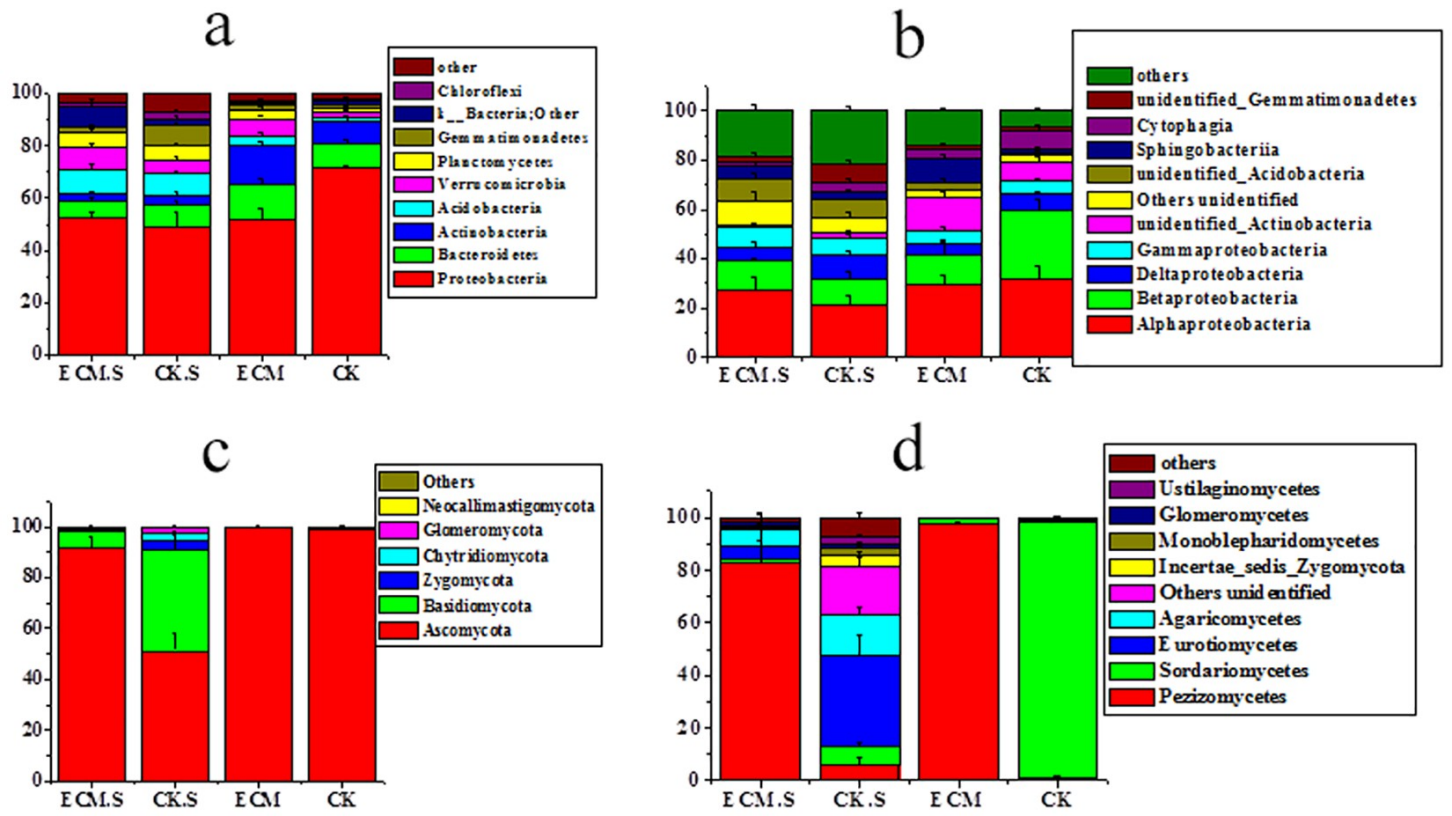
Taxonomic composition of bacterial and fungal communities associated with *Pinus armandii* root tips and surrounding soils at the phylum and class levels. ECM and ECM.S, ectomycorrhizae (*Pinus armandii* mycorrhized with *Tuber indicum*) and ectomycorrhizosphere soil. CK and CK.S, roots and soils from cultivated *P*. *armandii* without *T*. *indicum* partner. a, bacterial phyla; b, bacterial classes; c, fungal phyla; d, fungal classes. All experiments were conducted in triplicate.

Each bacterial 16S rRNA gene sequence was taxonomically assigned from the phylum level to the species level based on RDP 3 classifier. A total of 41 bacterial phyla were identified, out of which 23 were identified in all 12 samples (Fig 3a). Two archaeal phyla, Thaumarchaeota and Woesearchaeota were detected from the samples, which accounted for less than 0.1% of all bacteria associated with *P*. *armandii* roots. Five bacterial phyla, Proteobacteria (48.71%–71.59%), Bacteroidetes (6.45%–13.87%), Actinobacteria (4.00%–14.88%), Acidobacteria (1.45%–9.14%) and Verrucomicrobia (1.90%–8.26%) occupied the dominant position in all samples. Verrucomicrobia were found in greatest abundance in ECM and ECM.S than in other samples (P <0.05). The relative abundance of Gemmatimonadetes was significantly lower in ECM.S than in CK.S (P < 0.05). ECM contained more Proteobacteria than CK (P < 0.05).

Among the 81 bacterial classes detected, Alphaproteobacteria (21.28%– 31.65%), Betaproteobacteria (10.46%– 27.88%), Deltaproteobacteria (4.54%– 9.79%) and Gammaproteobacteria (5.24%– 7.84%) were the most abundant (Fig 3b). ECM and ECM.S contained less Deltaproteobacteria than CK and CK.S. The relative abundance of Deltaproteobacteria, Cytophagia and Phycisphaerae in ECM.S was lower compared with CK.S, and Acidimicrobiia was more abundant in ECM.S compared with it in CK.S (P < 0.05). Betaproteobacteria in CK was more abundant than in ECM, whereas Phycisphaerae was more abundant in ECM than in CK (P < 0.05).

**Fig 4:**
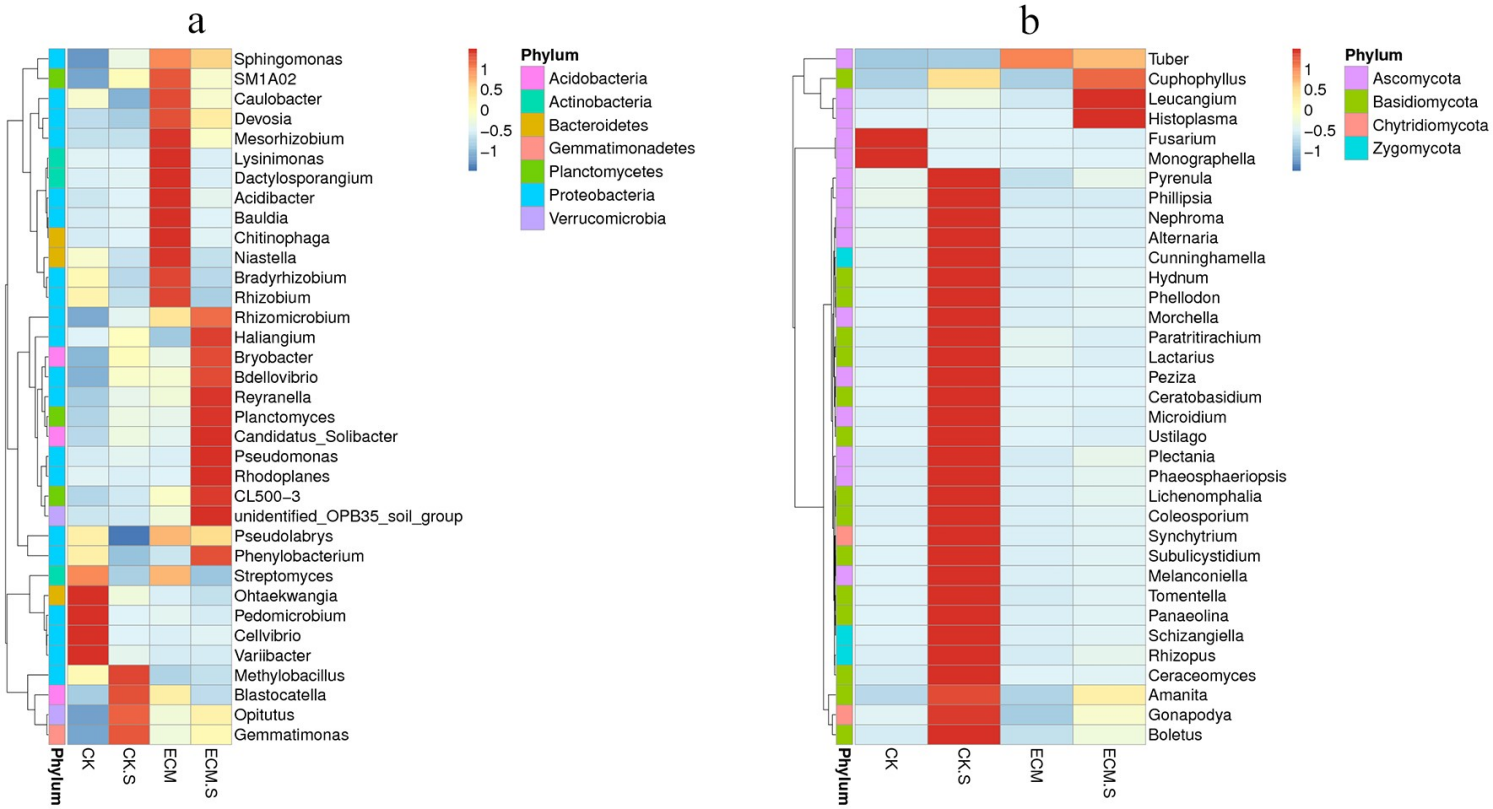
Heat-map analysis of the 35 most abundant bacterial and fungal genera in *Pinus armandii* roots and surrounding soils. ECM and ECM.S, ectomycorrhizae (*Pinus armandii* in association with *Tuber indicum*) and ectomycorrhizosphere soil. CK and CK.S, roots and soils from cultivated *P*. *armandii* without *T*. *indicum* partner. a, bacterial genera; b, fungal genera. The relative abundance of the sample at genus level increased with the increase of the color block value.

190 of the 439 genera were identified in all samples (Fig 4a). Among these, the dominant genera in all samples were *Streptomyces* (average 3.45%), *Pseudolabrys* (2.08%), *Opitutus* (1.65%), *Pseudomonas* (1.55%), *Ohtaekwangia* (1.44%), *Dactylosporangium* (1.44%) and *Mesorhizobium* (1.29%). *Mesorhizobium*, *Reyranena*, *Rhizomicrobium* and *Nordella* were significantly more abundant in ECM.S than in CK.S (P < 0.05). *Pseudomonas* was more abundant in ECM compared with CK (P < 0.05).

### Taxonomic analyses of fungal communities

A total of six phyla were observed in soil and root samples (Fig 3c). The relative abundance of two phyla, Ascomycota (average 85.21%) and Basidiomycota (11.72%), were dominant in all samples, however Basidiomycota was significantly more abundant in CK than in ECM (P < 0.05).

At the class level, Pezizomycetes (average 46.82%), Sordariomycetes (26.79%), Eurotiomycetes (10.03%), Agaricomycetes (5.61%) and Incertaesedis Zygomycota (1.13%) were the dominant taxa in all samples, of the 21 classes detected (Fig 3d). Pezizomycetes was significantly more abundant in ECM.S compared with CK.S (P < 0.05). ECM contained more Pezizomycetes and fewer Sordariomycetes than CK (P < 0.05).

A total of 122 genera were observed, and 27 of these were detected in all samples (Fig 4b). The most abundant observed genera were *Tuber* (average relative abundance of 89.28% in ECM and ECM.S), *Monographella* (average 7.48%), *Cuphophyllus* (2.04%) and *Melanconiella* (1.34%). *Tuber* was only detected in soil and *P*. *armandii* roots inoculated with *T*. *indicum*. *Cuphophyllus*, *Melanconiella* and *Gonapodya* were not observed in the ECM treatment.

### UniFrac analysis

**Fig 5:**
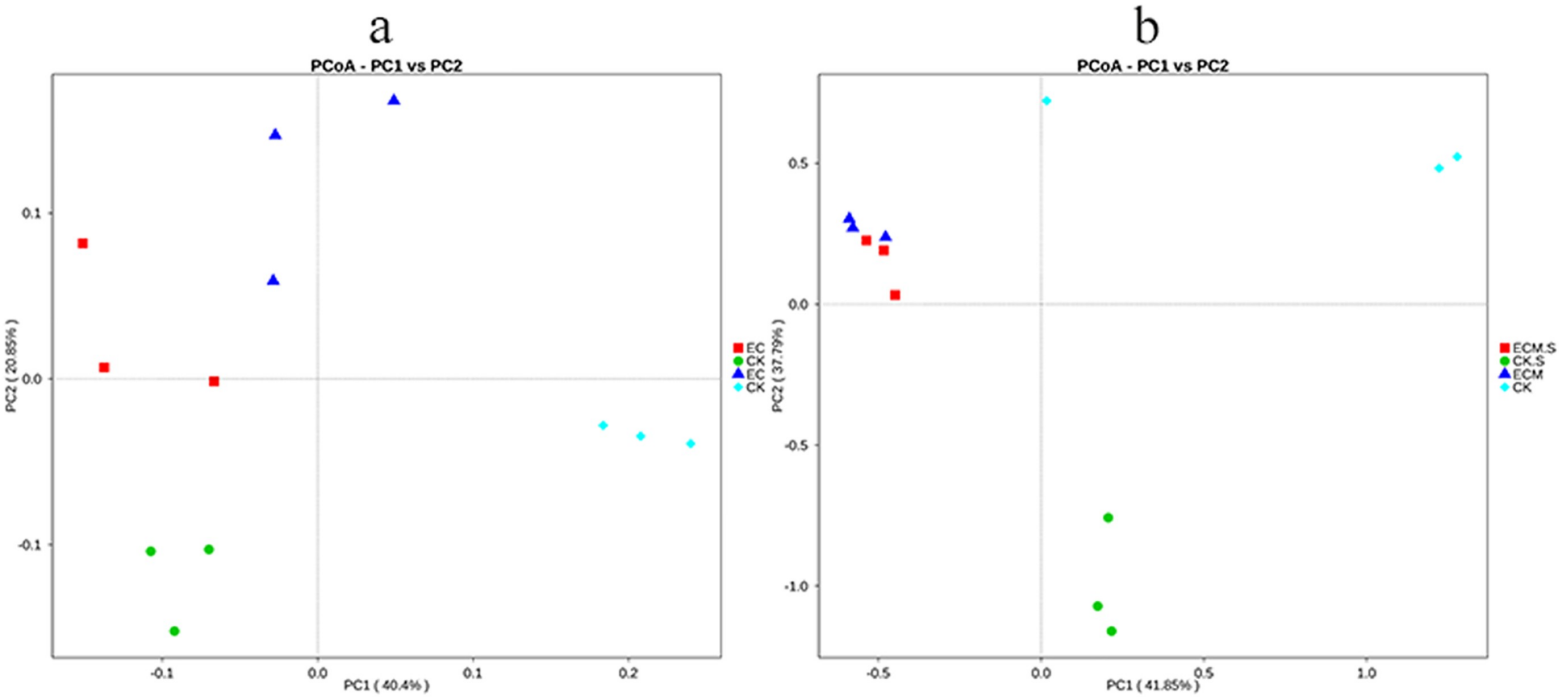
Principal Coordinate Analysis (PCoA) of bacterial (a) and fungal (b) communities associated with *Pinus armandii* roots and surrounding soils with or without *Tuber indicum* partner based on weighted UniFrac analysis. ECM and ECM.S, ectomycorrhizae (*Pinus armandii* mycorrhized with *Tuber indicum*) and ectomycorrhizosphere soil. CK and CK.S, roots and soils from cultivated *P*. *armandii* without *T*. *indicum* partner.

The differences in bacterial and fungal communities between the samples were estimated using UniFrac analysis (Fig 5). In the samples containing *T*. *indicum* in association with *P*. *armandii*, the associating bacterial community structure was significantly different compared with the control treatments, implying that the ectomycorrhizal fungi (ECMF) can affect the structure of the microbial community on its host and in the surrounding rhizosphere soil.

### Correlation analysis between microbial community and soil properties

There were significant correlations between some soil properties and the diversity of microbial communities (S1 Table). Clay content was positively correlated with observed bacterial species in soil according to the Shannon, Simpson, Chao1 and ACE indices. Total phosphorus and available phosphorus were negatively correlated with observed bacterial species and the Shannon index. Available zinc content was negatively correlated with bacteria in soil according to the Shannon and Simpson indices, whereas zinc was positively correlated with bacteria in the root tip according to the Chao1 and ACE indices. Effective nitrogen, available manganese, available copper and available magnesium were positively correlated with bacterial diversity according to the Simpson index in roots from both the control and ectomycorrhizae treatments.

Effective nitrogen and available copper were negatively correlated with soil fungi according to the Shannon index, observed fungal species and the Shannon index (S2 Table). Fungal species in root tips were negatively correlated with organic matter, available phosphorus, total potassium and available manganes according to some diversity indices, such as the observed species, Shannon, Simpson, Chao1 and ACE indices.

## Discussion

Using Hiseq sequencing, we analyzed the effects of *T*. *indicum* ectomycorrhizal association on the microbial communities of *Pinus armandii* and the surrounding ectomycorrhizosphere soil during early symbiotic stage. When *T*. *indicum* was present it was dominant (80.91% in ectomycorrhizosphere soil and 97.64% in ectomycorrhizae) reducing bacterial and fungal diversity in the root and in the surrounding soil (Tables 1 and 2). *Proteobacteria* was more abundant in the ectomycorrhizal soil compared with the control soil, indicating that this group closely related to the presence of truffle mycorrhiza, may play a role in the ectomycorrhizal synthesis. UniFrac analysis indicated that *T*. *indicum* directly or indirectly affected the microbial communities surrounding it, which initially come from the surrounding environment, such as air or water, and were finally significantly different from the communities of the control (Fig 5).

Previous work has shown evidence for reduced biodiversity of bacteria and fungi in the brûlé area in cultivated or wild conditions [15, 16, 19, 50]. Similarly, a reduced biodiversity of bacteria and fungi communities were observed in the ectomycorrhizosphere soil of *P*. *armandii* even in the seedling stage. However, the diversity of ectomycorrhizal bacterial diversity was improved to some extent compared with the non-ectomycorrhizae, especially the Simpson index (P < 0.05), which indicated that the truffle mycelia exert specific effects on the surrounding microbial communities. Previous study[39] found that α-Proteobacteria affiliated with *Sinorhizobium*, *Rhizobium* and *Bradyrhizobium* spp and γ-Proteobacteria, mostly fluorescent pseudomonads, were predominant components of truffle bacterial communities. Our results also indicated a more abundant *Bradyrhizobium*, *Rhizobium* and *Pseudomonas* communities existing in ectomycorrhizosphere soil and ectomycorrhizae compared with the CK and CK.S, indicating that these species are closely related to the presence of truffle mycelia and may play an important role in the growth and mycorrhizal synthesis of truffles. Previous study indicated that *T*. *melanosporum* ascocarps selected specific bacterial communities from the surrounding soil which may contribute to the development, maturation and even aroma of the Black truffle [22, 51]. Our study also showed that the *T*. *indicum* shaped the microbial communities of ectomycorhizosphere soil and ectomycorrhizae during the colonization of *P*. *armandii* even in the symbiotic stage.

Despite the reduced diversity of bacteria and fungi in ectomycorrhizosphere soil and ectomycorrhizae compared to the control samples, some bacterial genera, such as *Reyranena*, *Rhizomicrobium*, *Nordella* and *Pseudomonas* and some fungal genera, such as *Cuphophyllus*, *Leucangium* and *Histoplasma* were significantly enriched in ectomycorrhizosphere soil and ectomycorrhizae compared with CK.S and CK. Previous studies showed that combination of *Pseudomonas fluorescens* and *T*. *melanosporum* improved the establishment and functioning of ectomycorrhizal symbiosis [23]. *Pseudomonas* was more abundant in ectomycorrhizosphere soil. However, it was clear that the dominance of truffle mycelia did reduce the diversity and composition of endophytic pathogenic fungi, such as *Fusarium*, *Monographella*, *Ustilago* and *Rhizopus* and other competitive mycorrhizal fungi, such as *Amanita*, *Lactarius* and *Boletus*, supporting previous work that suggests ectomycorrhizal fungi can have a protective effect on the growth of plant host [52–54], for example, by reducing the infection of plants by microbes.

*T*. *indicum* mycelia were dominant (80.91%– 97.64%) among the fungal communities in the ectomycorrhizosphere and ectomycorrhizae, which was consistent with other studies. Whereas in another study, *T*. *magnatum* ectomycorrhiza seem to be absent or very rare when its ascomata formed and other mycorrhizal fungi were co-localized in the same root tips [20, 55]. This discrepancy may be due to the different survival strategies of truffle in different growth periods. Thus we hypothesized that during the early symbiotic stage, truffles compete with other mycorrhizal fungi for nutrition from the host tree. Whereas during the period when truffles are forming ascocarps, the ecological niche may be occupied by other mycorrhizal fungi owing to the absence of truffle mycelia. This strategy may help the ascocarpous produce of truffle, since it cannot be excluded that truffle may form other types of symbiosis, such as orchid-like mycorrhizas [20], revealed for other truffle species or other mycorrhizal fungi played a certain role in truffle ascocarpous produce.

Hierarchical cluster analysis of the similarities between rhizosphere and ectomycorrhizosphere soil based on the soil properties differentiated significantly (Fig 2), indicating that the mycorrhizal synthesis had a feedback effect on soil properties, which was consistent with other studies [56]. Some of the available nutrients and mineral elements, such as the organic matter, effective nitrogen, available phosphorus, available manganese, available calcium and available magnesium, were slightly higher in ectomycorrhizosphere soil during symbiotic stage than CK.S. The differences observed in soil properties may contribute to the synthesis of truffle ectomycorrhizas [30, 56–58], since the important effects of soil properties, such as pH and carbonate content, on “black truffle” (*Tuber melanosporum*) production are well known. Compared with the black truffle *T*. *melanosporum* and the Italian white truffle *T*. *magnatum*, which are more suitable for alkalescence soil [11, 57], *T*. *indicum* is often found in slightly acidic soil [59]. Meanwhile, we found that some soil properties had significant correlations with bacterial and fungal diversity in soil or root tips. This work illustrates the interactive network that exists among ectomycorrhizal fungi, soil properties and microbial communities and the host plant

Successful ectomycorrhizal synthesis is the basis of efficient cultivation [1, 4, 5]. In truffle orchards, several factors have been found to affect the ectomycorrhizal communities, such as the age of the plantation, the host species, productivity, the surrounding environment and management [20]. Different experimental situations and methodologies make it difficult to compare the communities of truffle-associated microbes and to properly explain spatial and temporal microbial dynamics as well as their relationship with ascocarp production [11, 16]. Knowledge of microbial communities of surrounding soil of ectomycorrhizosphere and plant roots during early symbiotic stage may significantly improved our knowledge about ecological effect and tentative life strategies of truffles. Meanwhile, the application of high-throughput sequencing technology serves to broaden our knowledge of the composition of microbial communities surrounding the ectomycorrhizae, as most microbes in nature are unculturable. Further studies are needed to understand the interactions between truffles and other organisms in the rhizosphere, including mycorrhization helper bacteria (MHB). MHB are promising for establishing truffle plantations and should be investigated.

## Supporting information

S1 Fig Rarefaction curves for bacterial (a) and fungal (b) Operational Taxonomic Units (OTUs) in different samples (cut-off value at 97% similarity). In the rarefaction curves, the number of OTUs increased with sequencing reads. ECM and ECM.S, ectomycorrhizae (*Pinus armandii* mycorrhized with *Tuber indicum*) and ectomycorrhizosphere soil. CK and CK.S, roots and soils from cultivated *P*. *armandii* without *T*. *indicum* partner.
(DOCX)

S2 Fig Numbers of shared and specific bacterial (a) and fungal (b) Operational Taxonomic Units (OTUs). ECM and ECM.S, ectomycorrhizae (*Pinus armandii* in association with *Tuber indicum*) and ectomycorrhizosphere soil. CK and CK.S, roots and soils from cultivated *P*. *armandii* without *T*. *indicum* partner.
(TIF)

S1 Table Spearman correlation coefficient (rs) between soil properties and indicators of bacterial community structure. (DOCX)

S2 Table Spearman correlation coefficient (rs) between soil properties and indicators of fungal community structure. (DOCX)
