# Supplementary material for: Tuber indicum shapes the microbial communities of ectomycorhizosphere soil and ectomycorrhizae of an indigenous tree (Pinus armandii)
Source: PLoS One. 2017 Apr 14;12(4):e0175720. doi: 10.1371/journal.pone.0175720 (PMC5391931; doi:10.1371/journal.pone.0175720)
Supplement: S1 Fig — In the rarefaction curves, the number of OTUs increased with sequencing reads. ECM and ECM.S, ectomycorrhizae (Pinus armandii mycorrhized with Tuber indicum) and ectomycorrhizosphere soil. CK and CK.S, roots and soils from cultivated P. armandii without T. indicum partner. (DOCX) [file pone.0175720.s001.docx]

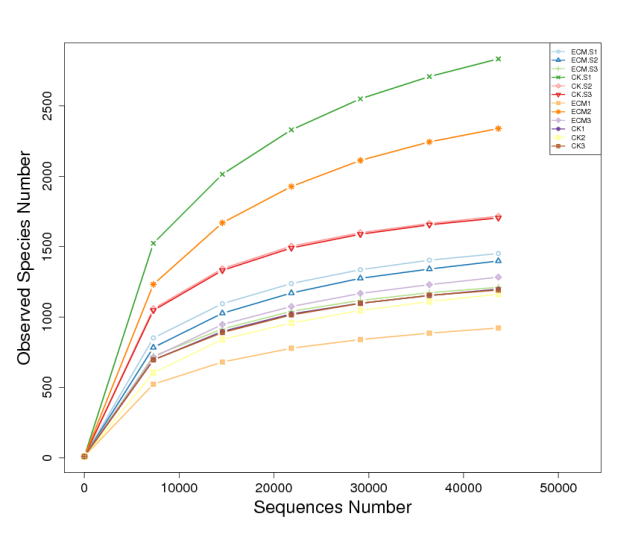

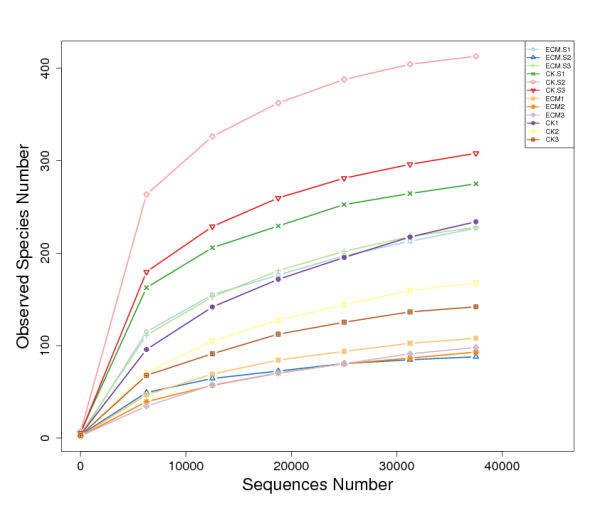


**b**

**a**

Fig. A1 Rarefaction curves for bacterial (a) and fungal (b)operational taxonomic units (OTUs) in different samples (cut-off value at 97% similarity). In the rarefaction curves, the number of OTUs increased with sequencing reads. ECM and ECM.S, ectomycorrhizae (*Pinus armandii* mycorrhized with *Tuber indicum*) and ectomycorrhizosphere soil. CK and CK.S, roots and soils from cultivated *P. armandii* without *T. indicum* partner.
