# Supplementary figures and images for: Tuber indicum shapes the microbial communities of ectomycorhizosphere soil and ectomycorrhizae of an indigenous tree (Pinus armandii)
Source: PLoS One. 2017 Apr 14;12(4):e0175720. doi: 10.1371/journal.pone.0175720 (PMC5391931; doi:10.1371/journal.pone.0175720)

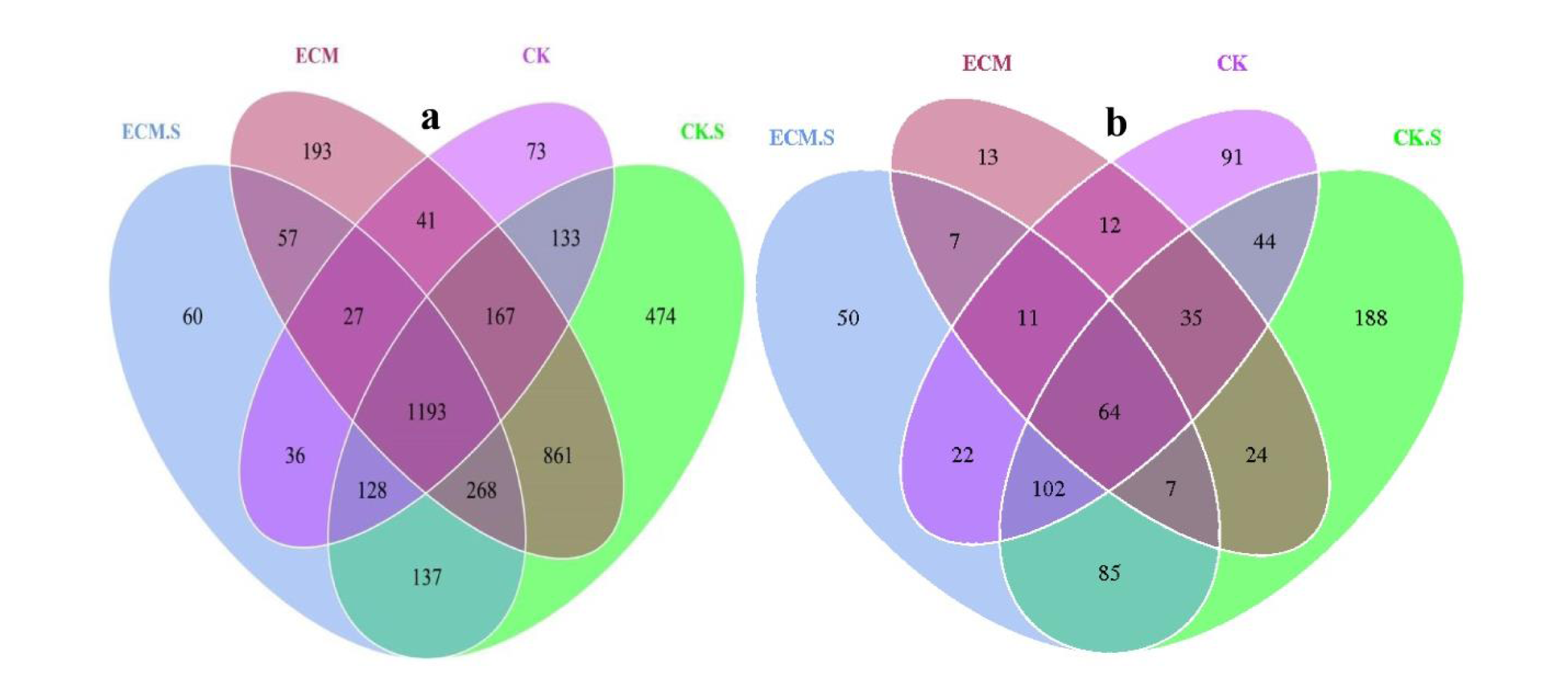

Supplement: S2 Fig — ECM and ECM.S, ectomycorrhizae (Pinus armandii in association with Tuber indicum) and ectomycorrhizosphere soil. CK and CK.S, roots and soils from cultivated P. armandii without T. indicum partner. (TIF) [file pone.0175720.s002.tif]
