# Supplementary material for: Tuber indicum shapes the microbial communities of ectomycorhizosphere soil and ectomycorrhizae of an indigenous tree (Pinus armandii)
Source: PLoS One. 2017 Apr 14;12(4):e0175720. doi: 10.1371/journal.pone.0175720 (PMC5391931; doi:10.1371/journal.pone.0175720)
Supplement: S1 Table — (DOCX) [file pone.0175720.s003.docx]

**S3 Table. Spearman correlation coefficient (rs) between soil properties and indicators of bacterial community structure.**

| Soil properties | Soil | | | | | | | Root tip | | | | | | |
| --- | --- | --- | --- | --- | --- | --- | --- | --- | --- | --- | --- | --- | --- | --- |
|  | Observed species | Shannon | Simpson | Chao1 | ACE | Goods coverage | PD whole tree | Observed species | Shannon | Simpson | Chao1 | ACE | Goods coverage | PD whole tree |
| pH | 0.23 | 0.14 | 0.10 | 0.25 | 0.25 | -0.32 | 0.17 | -0.08 | 0.28 | 0.42 | -0.10 | -0.11 | 0.24 | 0.06 |
| Sand (%) | -0.53 | -0.70 | -0.60 | -0.49 | -0.48 | 0.34 | -0.56 | 0.03 | 0.33 | 0.79 | 0.05 | 0.06 | -0.06 | 0.02 |
| Silt (%) | -0.47 | -0.35 | -0.35 | -0.49 | -0.49 | 0.55 | -0.45 | 0.40 | 0.16 | -0.19 | 0.40 | 0.40 | -0.48 | 0.32 |
| Clay (%) | 0.87* | 0.92** | 0.83* | 0.85* | 0.85* | -0.78 | 0.88* | -0.37 | -0.43 | -0.51 | -0.41 | -0.41 | 0.48 | -0.30 |
| OM (g/kg) | -0.77 | -0.86* | -0.77 | -0.74 | -0.73 | 0.62 | -0.78 | 0.32 | 0.48 | 0.75 | 0.35 | 0.35 | -0.38 | 0.31 |
| TN (g/kg) | -0.20 | -0.40 | -0.41 | -0.16 | -0.15 | 0.05 | -0.20 | 0.13 | 0.19 | 0.48 | 0.15 | 0.16 | -0.19 | 0.09 |
| TP (g/kg) | -0.83* | -0.93** | -0.93** | -0.80 | -0.80 | 0.72 | -0.85* | 0.64 | 0.67 | 0.59 | 0.67 | 0.66 | -0.71 | 0.59 |
| TK (g/kg) | -0.68 | -0.83* | -0.70 | -0.64 | -0.64 | 0.51 | -0.74 | -0.02 | 0.36 | 0.77 | 0.00 | -0.01 | 0.01 | -0.05 |
| AN (mg/kg) | -0.24 | -0.53 | -0.51 | -0.19 | -0.18 | 0.01 | -0.32 | 0.01 | 0.46 | 0.89* | 0.02 | 0.02 | 0.05 | 0.05 |
| AP (mg/kg) | -0.91* | -0.82* | -0.68 | -0.90* | -0.89* | 0.84* | -0.90* | 0.33 | 0.50 | 0.66 | 0.36 | 0.35 | -0.37 | 0.39 |
| AK (mg/kg) | 0.10 | 0.34 | 0.52 | 0.06 | 0.05 | 0.04 | 0.10 | -0.70 | -0.62 | -0.47 | -0.71 | -0.72 | 0.71 | -0.70 |
| AFe (mg/kg) | -0.61 | -0.52 | -0.30 | -0.59 | -0.59 | 0.52 | -0.62 | -0.24 | 0.14 | 0.65 | -0.22 | -0.23 | 0.25 | -0.15 |
| AMn (mg/kg) | -0.64 | -0.84* | -0.72 | -0.60 | -0.59 | 0.44 | -0.71 | 0.01 | 0.41 | 0.84* | 0.04 | 0.03 | -0.02 | -0.01 |
| ACu (mg/kg) | -0.24 | -0.58 | -0.65 | -0.19 | -0.17 | 0.00 | -0.33 | 0.32 | 0.73 | 0.95** | 0.32 | 0.32 | -0.23 | 0.37 |
| AZn (mg/kg) | -0.65 | -0.84* | -0.92** | -0.61 | -0.60 | 0.51 | -0.67 | 0.80 | 0.79 | 0.64 | 0.83* | 0.83* | -0.86* | 0.76 |
| ACa (cmol/kg) | -0.34 | -0.58 | -0.61 | -0.29 | -0.28 | 0.16 | -0.36 | 0.36 | 0.47 | 0.67 | 0.39 | 0.39 | -0.40 | 0.34 |
| AMg (cmol/kg) | -0.58 | -0.80 | -0.73 | -0.54 | -0.53 | 0.38 | -0.66 | 0.12 | 0.56 | 0.89* | 0.13 | 0.12 | -0.08 | 0.13 |

OM, organic matter; TN, total nitrogen; TP, total phosphorus; TK, total potassium; AN, effective nitrogen; AP, available phosphorus; AK, available potassium; AFe, available iron; AMn, available manganese; ACu, available copper; AZn, available zinc; ACa, available calcium;AMg, available magnesium.

*Significant at p < 0.05; **Significant at p < 0.01.

Chao1, estimator of richness.
