# Supplementary material for: Tuber indicum shapes the microbial communities of ectomycorhizosphere soil and ectomycorrhizae of an indigenous tree (Pinus armandii)
Source: PLoS One. 2017 Apr 14;12(4):e0175720. doi: 10.1371/journal.pone.0175720 (PMC5391931; doi:10.1371/journal.pone.0175720)
Supplement: S2 Table — (DOCX) [file pone.0175720.s004.docx]

**S4 Table. Spearman correlation coefficient (rs) between soil properties and indicators of fungal community structure.**

| Soil properties | Soil | | | | | | | Root tip | | | | | | |
| --- | --- | --- | --- | --- | --- | --- | --- | --- | --- | --- | --- | --- | --- | --- |
|  | Observed species | Shannon | Simpson | Chao1 | ACE | Goods coverage | PD whole tree | Observed species | Shannon | Simpson | Chao1 | ACE | Goods coverage | PD whole tree |
| pH | -0.47 | -0.60 | -0.56 | -0.36 | -0.35 | -0.27 | -0.36 | -0.01 | -0.01 | -0.10 | 0.03 | 0.05 | -0.30 | 0.23 |
| Sand (%) | -0.50 | -0.70 | -0.78 | -0.38 | -0.39 | -0.11 | -0.45 | -0.61 | -0.78 | -0.78 | -0.60 | -0.59 | 0.32 | -0.62 |
| Silt (%) | 0.12 | 0.30 | 0.45 | -0.01 | -0.01 | 0.16 | 0.06 | -0.32 | -0.13 | 0.04 | -0.30 | -0.31 | 0.52 | -0.39 |
| Clay (%) | 0.32 | 0.34 | 0.26 | 0.33 | 0.34 | -0.05 | 0.34 | 0.81 | 0.79 | 0.64 | 0.78 | 0.78 | -0.74 | 0.88* |
| OM (g/kg) | -0.56 | -0.69 | -0.73 | -0.48 | -0.50 | 0.10 | -0.50 | -0.82* | -0.83* | -0.72 | -0.83* | -0.83* | 0.61 | -0.87* |
| TN (g/kg) | -0.34 | -0.38 | -0.49 | -0.28 | -0.30 | 0.31 | -0.35 | -0.25 | -0.37 | -0.35 | -0.27 | -0.28 | 0.06 | -0.36 |
| TP (g/kg) | -0.54 | -0.45 | -0.29 | -0.57 | -0.58 | 0.26 | -0.56 | -0.83* | -0.70 | -0.49 | -0.79 | -0.79 | 0.68 | -0.86* |
| TK (g/kg) | -0.41 | -0.59 | -0.51 | -0.33 | -0.31 | -0.45 | -0.39 | -0.71 | -0.88* | -0.91* | -0.64 | -0.61 | 0.43 | -0.62 |
| AN (mg/kg) | -0.67 | -0.82* | -0.79 | -0.54 | -0.53 | -0.21 | -0.62 | -0.44 | -0.61 | -0.68 | -0.37 | -0.35 | -0.03 | -0.30 |
| AP (mg/kg) | -0.52 | -0.74 | -0.78 | -0.44 | -0.47 | -0.04 | -0.41 | -0.95** | -0.86* | -0.70 | -0.98** | -0.98** | 0.85* | -0.96** |
| AK (mg/kg) | 0.67 | 0.46 | 0.42 | 0.69 | 0.72 | -0.78 | 0.69 | 0.26 | 0.09 | -0.08 | 0.26 | 0.27 | 0.00 | 0.30 |
| AFe (mg/kg) | -0.34 | -0.77 | -0.92** | -0.16 | -0.17 | -0.51 | -0.18 | -0.68 | -0.81 | -0.85* | -0.71 | -0.70 | 0.52 | -0.61 |
| AMn (mg/kg) | -0.50 | -0.68 | -0.62 | -0.40 | -0.39 | -0.36 | -0.47 | -0.71 | -0.88* | -0.91* | -0.64 | -0.62 | 0.37 | -0.63 |
| ACu (mg/kg) | -0.88* | -0.89* | -0.75 | -0.79 | -0.78 | -0.01 | -0.84* | -0.49 | -0.53 | -0.53 | -0.40 | -0.38 | -0.04 | -0.30 |
| AZn (mg/kg) | -0.70 | -0.53 | -0.38 | -0.75 | -0.76 | 0.55 | -0.73 | -0.73 | -0.54 | -0.31 | -0.69 | -0.70 | 0.50 | -0.76 |
| ACa (cmol/kg) | -0.60 | -0.59 | -0.63 | -0.54 | -0.56 | 0.39 | -0.59 | -0.45 | -0.48 | -0.40 | -0.45 | -0.46 | 0.16 | -0.51 |
| AMg (cmol/kg) | -0.63 | -0.76 | -0.62 | -0.54 | -0.52 | -0.37 | -0.59 | -0.70 | -0.81 | -0.83* | -0.61 | -0.58 | 0.31 | -0.54 |

OM, organic matter; TN, total nitrogen; TP, total phosphorus; TK, total potassium; AN, effective nitrogen; AP, available phosphorus; AK, available potassium; AFe, available iron; AMn, available manganese; ACu, available copper; AZn, available zinc; ACa, available calcium; AMg, available magnesium.

*Significant at p < 0.05; **Significant at p < 0.01.

Chao1, estimator of richness.
